# The Use of Bi-Potentiostat as a Simple and Accurate Electrochemical Approach for the Determination of Orthophosphate in Seawater

**DOI:** 10.3390/s23042123

**Published:** 2023-02-13

**Authors:** Mahmoud Fatehy Altahan, Mario Esposito, Boie Bogner, Eric P. Achterberg

**Affiliations:** 1 Chemical Oceanography Department, GEOMAR Helmholtz Centre for Ocean Research, 24148 Kiel, Germany; 2 Central Laboratory for Environmental Quality Monitoring, National Water Research Centre, El-Qanater El-Khairia 13621, Egypt

**Keywords:** Bi-potentiostat, electrochemical sensor, orthophosphate sensor, Python programming, phosphomolybdate complex method

## Abstract

Autonomous *on-site* monitoring of orthophosphate (PO_4_^3−^), an important nutrient for primary production in natural waters, is urgently needed. Here, we report on the development and validation of an *on-site* autonomous electrochemical analyzer for PO_4_^3−^ in seawater. The approach is based on the use of flow injection analysis in conjunction with a dual electrochemical cell (i.e., a bi-potentiostat detector (FIA-DECD) that uses two working electrodes sharing the same reference and counter electrode. The two working electrodes are used (molybdate/carbon paste electrode (CPE) and CPE) to correct for matrix effects. Optimization of squarewave voltammetry parameters (including step potential, amplitude, and frequency) was undertaken to enhance analytical sensitivity. Possible interferences from non-ionic surfactants and humic acid were investigated. The limit of quantification in artificial seawater (30 g/L NaCl, pH 0.8) was 0.014 µM for a linear concentration range of 0.02–3 µM. The system used a Python script for operation and data processing. The analyzer was tested for ship-board PO_4_^3−^ determination during a four-day research cruise in the North Sea. The analyzer successfully measured 34 samples and achieved a good correlation (Pearson’ R = 0.91) with discretely collected water samples analyzed using a laboratory-based colorimetric reference analyzer.

## 1. Introduction

Phosphorus (P) is one of the key elements required for the functioning of marine ecosystems. Like nitrogen, P is an important macronutrient for all living organisms and is essential for primary production and thus control of atmospheric oxygen levels [1,2]. Phosphorus can be found in marine waters in dissolved and particulate forms. Orthophosphate, the major inorganic form of phosphorus, plays a critical structural and functional role in all marine organisms. Orthophosphate is released into waters following organic matter degradation via heterotrophic metabolic pathways and is available for either biological uptake, sorption onto mineral fractions, or incorporation into authigenic minerals (e.g., carbonate fluorapatite) [1,3]. High external P inputs into marine ecosystems, such as run-off from agricultural P fertilizers, may lead to eutrophication and subsequent oxygen depletion in affected coastal areas. In oligotrophic regions, which account for about 40% of the world’s oceans, PO_4_^3−^ concentrations are at nanomolar levels due to low supply and removal by primary production.

Since the early 1980s, research has been conducted into the development of online orthophosphate sensors [4]. Conventional laboratory methods based on classical colorimetric techniques are labor intensive and can only be used for the measurements of a limited number of discretely collected samples.

The Molybdenum Blue method is the most widely used laboratory technique for PO_4_^3−^. The method, first described by Murphy and Riley [5], is based on the reaction of orthophosphate with molybdate under acidic conditions to form a yellow phosphomolybdate complex, which is readily reduced to a blue colored complex by an excess of ascorbic acid in the presence of antimony (Sb) and organic acid (tartaric acid) in potassium antimony tartrate. The product complex is characterized by two spectral peaks at 720 nm and 880 nm, with a higher intensity at 880 nm, and the complex is stable for several hours. The Molybdenum Blue method is limited by the stability of ascorbic acid, whose lifetime under dark conditions is only two months [6].

The Molybdenum Yellow method was first described by Kriston and Mellon [7]. The method is based on the addition of excess molybdate to an acidic solution of vanadate and PO_4_^3−^ to form a yellow phosphomolybdate complex with a spectral peak at 340 nm. The reagent mixture is stable for about one year. Otherwise, the use of vanadium (V) as ammonium vanadate may have toxic effects on aquatic organisms [8].

A number of deployable sensors for PO_4_^3−^ in seawater has been reported based on the colorimetric Molybdenum Blue method. The Hydrocycle-PO4 sensor [9] is manufactured by Sea-Bird Scientific (Philomath, OR, USA), and the Lab-On-Chip (LOC) sensor is by Clearwater Sensor Ltd. (Southampton, UK) and based on microfluidic technology [10]. The WIZ sensor is manufactured by SYSTEA SpA (Anagni, Lazio, Italy) and is based on a patented micro-loop flow analysis (µLFA) [11]. The NuLAB sensor is manufactured by Green Eyes LLC (Easton, PA, USA) and is based on flow injection analysis (FIA) [12].

As reported for the NuLAB and LOC systems and in our recent work [13,14,15], an optical correction is performed in spectrophotometric sensors using a reference channel to give the sensor high applicability in a variety of natural waters with high precision and accuracy. For this purpose, the sample is passed through a reference channel to obtain the light intensity before reagents are added. This is very useful to correct for interferences in the sample matrix, including salinity variations and colored dissolved organic matter (CDOM).

The long-term use of wet chemical sensors is constrained by reagent availability and chemical stability of some of the chemicals used (e.g., ascorbic acid in the case of the Molybdenum Blue method). As an alternative, electrochemistry forms a good technique for miniaturized, ready-to-use sensors with a minimized reagent and energy consumption and may be well-suited for long-term environmental quality monitoring.

Several electrochemical sensors have been developed for the quantification of PO_4_^3−^ in seawater based on amperometric or voltammetric techniques. Here, an electrochemical force potential is applied to the electrode or the interface of a solution, causing a chemical reaction and, consequently, a current to flow, which is subsequently recorded [16]. The majority of the electrochemical probes for PO_4_^3−^ are based on molybdate chemistry and utilize a variety of electrochemical reactions.

A commonly used reaction involves the electrochemical reduction in the phosphomolybdate complex. This complex can be formed in situ via complexation between orthophosphate and ammonium molybdate, previously introduced into the solution. The determination of the phosphomolybdate complex is completed by cyclic voltammetry and allows quantification of the PO_4_^3−^ concentration [17].

Another type of reaction involves the self-production of molybdate ions in the electrochemical cell by direct electrolysis of a solid molybdenum electrode. The molybdate ions are then readily available for reaction with orthophosphate to form a phosphomolybdate complex. The complex formed was detected at the gold electrode by amperometry [18], squarewave voltammetry [19], and differential pulse voltammetry [20].

The second type of reaction is based on the reaction of orthophosphate with molybdate ions pre-charged on a screen-printed electrode (with a carbon ink working electrode containing 5% carbon black) and the presence of other reagents, including potassium chloride and sulfuric acid. The phosphomolybdate complex is formed after 150 s in the sample solution. The phosphomolybdate complex is then detected by cyclic voltammetry [21].

There are several reports in the literature on electroanalytical methods and promising prototypes for orthophosphate determination in seawater or freshwater. However, there are no reports of online deployment of these systems.

We recently reported on the determination of orthophosphate in seawater by square wave voltammetry on a carbon paste electrode modified with ammonium molybdate and pre-treated by cyclic voltammetry in sodium hydroxide [22].

Here, we describe for the first time the application of a bi-potentiostat (i.e., double electrochemical cell) which was recently reported for the efficient determination of dissolved gases [23], where two working electrodes are used based on carbon paste technology. The first working electrode contains the chemical reagent (molybdate reagent) in a carbon paste and the second working electrode has no additive in the carbon paste base and serves as a reference channel for matrix interference correction. The signal of the second working electrode is subtracted from that of the first working electrode. The bi-potentiostat approach was applied in a home-built analyzer for online determination of orthophosphate in seawater on a research vessel in the North Sea, using a Python script for automated data processing.

## 2. Materials and Methods

### 2.1. Chemicals

Reagents and calibration solutions used in this study were prepared with ultrapure water (resistivity 18.2 MΩ-cm, MilliQ, Millipore Water System, Burlington, MA, USA) and reagent-grade analytical salts. Glass and plastic wares were cleaned thoroughly before use. They were rinsed with ultrapure water and then immersed in an acid bath containing 10% (*v*/*v*) concentrated HCl (37%, reagent grade, Carl Roth, Germany) for >24 h and then rinsed again with ultrapure water.

Calibration solutions and standards for PO_4_^3−^ and H_4_SiO_4_ analyzes were prepared in artificial seawater. Artificial seawater with a salinity of 30 was prepared from sodium chloride (Sigma Aldrich, Burlington, MA, USA) at 30 g/L. For online acidification of seawater samples, 500 mL heat-resistant borosilicate glass laboratory bottles were filled with 50% H_2_SO_4_ (98%, Carl Roth).

The stock solution of PO_4_^3−^ (1 mM) was prepared by dissolving 0.136 g of potassium dihydrogen sulfate (KH_2_PO_4_, Merck, Kenilworth, NJ, USA) in 1000 mL of ultrapure water. The stock solution of H_4_SiO_4_ (1 mM) was prepared from sodium metasilicate pentahydrate (NaSiO_3_.5H_2_O, Sigma Aldrich) by dissolving 0.0212 g in 1000 mL of ultrapure water.

A stock solution of the surfactant Triton x-100 was prepared at 50% (*v*/*v*) (50 mL of Triton x-100 (Sigma Aldrich) and 50 mL of isopropanol alcohol (C_3_H_8_OH, Fischer Scientific, USA). A stock solution for humic acid was prepared by dissolving 0.1 g of solid humic acid (Sigma Aldrich) into 100 mL of a solution of 0.1 M NaOH and neutralized by 37% HCl to pH ~ 7.5.

All the stock solutions were stored in 500 mL high-density polyethylene (HDPE) bottles (Nalgene, Thermo Scientific, USA) and kept refrigerated (4 °C) when not in use. Cleaning (0.1 M NaOH), blank, and standard solutions were freshly prepared prior to use and stored in 150 mL HDPE Nalgene bottles.

### 2.2. Description of Apparatus

**Figure 1:**
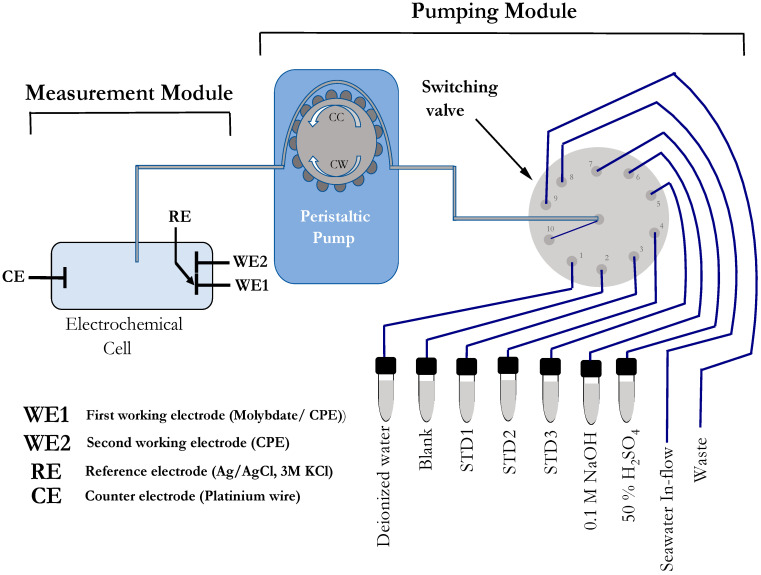
Schematic diagram of the FIA-DECD showing the peristaltic pump, electrochemical dual-channel cell, and switching valve with the connections for all reagents, standard solutions, and inflow of seawater.

A schematic diagram of the flow injection analyzer–dual electrochemical cell detector (FIA-DECD), used for PO_4_^3−^ analysis, is shown in Figure 1. The main hardware consists of two modules. The system is based on the application of continuous flow analysis. The solution is delivered by means of a peristaltic pump with the use of a multi-position switching valve [24]. The fluid transfer module consists of a peristaltic pump (DYNAMAX RP-1, Rainin Instrument Co., USA) that delivers liquids to the electrochemical cell and is connected to a 10-port valve (Cheminert C25Z series, VICI, TX, USA). Samples and reagents are delivered to or withdrawn from the electrochemical cell by adjusting the pump direction (clockwise (CW) or counter-clockwise (CC)), and switching the valve position. Both the peristaltic pump and the switching valve are controlled by a Python graphical user interface. The rotary fittings of the switching valve were equipped with finger-tight polyetheretherketone (PEEK) connectors to connect polytetrafluoroethylene (PTFE) tubing with an inner diameter of 0.8 mm for the transfer of standards and reagents. The same tubing and fittings were used for sample flow, connected to a Luer PEEK adapter (male–female) and a 0.45 µm syringe filter. For peristaltic liquid propulsion, pump tubing (Tygon LMT-55; green-green, inner diameter 1.85 mm) was used to deliver fluids to the electrochemical cell.

The measurement module consisted of a µStat 400 bi-potentiostat/galvanostat (Metrohm Dropsens, Spain) with 4 electrodes (2 working electrodes, reference electrode, counter electrode, and ground), a cable connection with alligator clips with USB type-B connectors, and RS -232 cables. All electrochemical measurements were performed using Dropview software (Metrohm). The electrochemical cell consisted of four electrodes: 2 working electrode holders for carbon paste (BASi, USA), a reference electrode of silver and silver chloride (Ag/AgCl) fed with saturated potassium chloride (3 M KCl), and a counter electrode of glassy carbon (BASi, US). A 50 mL glass beaker was used as an electrochemical cell and covered with parafilm. The synchronization between the measurement and fluid transfer modules was performed with an I/O configuration (i.e., RS 232) with the bi-potentiostat and an Arduino board, which was preconfigured to control the states of the peristaltic pump and exchange the digital signals with the bi-potentiostat. During the pumping step, the Arduino board was able to continuously send digital signals to the bi-potentiostat, and during the measurement step, the bi-potentiostat was able to send signals to the Arduino board to stop pumping.

### 2.3. Preparation of Modified Electrodes

The preparation of the molybdate/carbon paste electrode (CPE) and the CPE was carried out in several steps. First, the CPE holders were cleaned by placing them in plastic tubes filled with ethanol (CH_3_CH_2_OH ≥ 99.8%, ROTH, Germany) and sonicated at 30 °C for 90 min in an ultrasonic bath. Then, the carbon paste mixtures were prepared as follows: for the molybdate/CPE, 0.6 g of graphite powder (particle size < 20, Sigma Aldrich) and 0.1 g of ammonium molybdate tetrahydrate [(NH_4_)_6_Mo_7_O_24_.4H_2_O] (≥99%, Sigma Aldrich) were mixed in a mortar with 0.3 g paraffin oil (Sigma Aldrich) for 10 min using a pestle to form a uniform carbon paste. The paste was stored in a 5 mL tube (Eppendorf) until use. A small amount of paste was pressed into the cavity at the end of the electrode holder to produce the modified electrode. Later, an excess of the electrode material was removed by polishing using a piece of filter paper. Then, the modified electrode was rinsed with ultrapure water and inserted into the electrochemical cell. The carbon paste mixture for the CPE was prepared by mixing 0.7 g of graphite and 0.3 g of paraffin oil, and the same procedure as outlined above was used. The CPE was inserted into the electrochemical cell. Two electrochemical circuits were set up, where the two working electrodes, molybdate/CPE and CPE, shared the same auxiliary electrode and reference electrode and were immersed in a 50 mL glass vessel filled with a solution of 0.1 M NaOH; cyclic voltammetry was performed with a potential scan from −0.5 to 0.5 V at a scan rate of 0.1 Vs^−1^ for 10 scan cycles. This step is important to facilitate the formation of MoO_4_^2−^, which then reacts with orthophosphate ions to form a phosphomolybdate complex. Ultrapure water was introduced into the electrochemical cell for purification, followed by the injection of a blank solution of artificial seawater.

### 2.4. Data Processing

**Figure 2:**
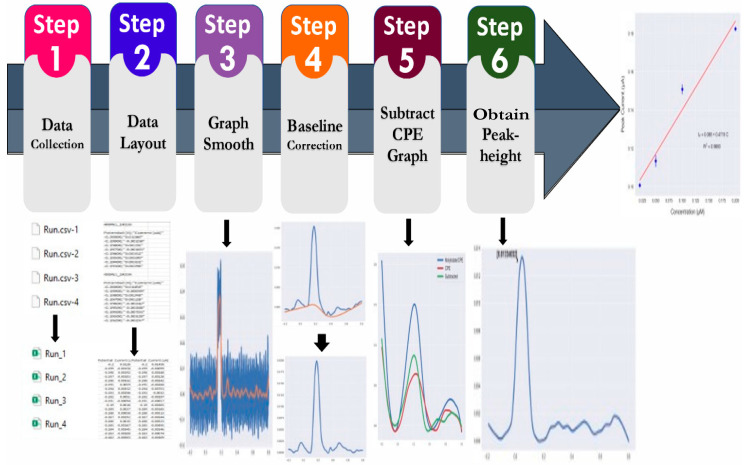
Flow chart for automatic data processing of raw data obtained using the Metrohm Dropview software.

Python software 3.9 was used to process the raw data obtained from the Dropview software 8400. Figure 2 shows the flowchart for the steps of the protocol. The protocol starts with reading the raw data obtained via the script editor in Dropview and producing the voltammograms of the two channels as columns (x, y) in an Excel spreadsheet, where x is the current (µA) and y the potential (V). The code is based on reading the files as column-separated values (csv) (**step 1**) and dividing them into individual columns. The columns (potential (V), current (µA)) are divided into two parts; the first part is for the molybdate/CPE (working electrode 1) and the second part is for the CPE (working electrode 2) (**step 2**). Then, the two parts are merged into one csv spreadsheet. Smoothing of the resulting peak was performed by nonparametric regression and locally weighted scatterplot smoothing (LOWESS) with a hyperparameter value of 0.04 (**step 3**). This hyperparameter value is the fraction that controls the local data window width, and the value of 0.04 makes the model more sensitive to the local data. Baseline correction was performed for background current subtraction using the PeakUtils Python package [25] (**step 4**); this package was used for peak quantification for each set of data with the indexes function. Subtraction of the CPE current from the molybdate/CPE current was performed (**step 5**) with subsequent determination of the peak height in µA using the SciPy Python module [26,27], which can detect peaks in the voltammogram (**step 6**). The obtained results were converted to concentrations in µM by constructing a calibration plot with the current in µA (y-axis) and concentration of PO_4_^3−^ in µM (x-axis) using the linear regression model via the Python Statsmodels library [28]. Calculating the concentration of the unknown sample in µM was conducted using the following equation.

(1) $$Concentration\left(µM\right)=(I-B)/S$$

where I is the peak current in µA of the sample, B is the intercept coefficient of the linear fit in µA, and S is the slope coefficient of the linear fit in µA·µM^−1^.

### 2.5. Analytical Procedure

The measurement cycle for PO_4_^3−^ begins with a calibration consisting of a blank solution and three standard solutions with known PO_4_^3−^ concentrations pre-acidified to pH 0.8 with 50% (*v*/*v*) sulfuric acid (H_2_SO_4_ 98%, Carl Roth), followed by an analysis of seawater samples acidified online with 50% (*v*/*v*) H_2_SO_4_. For the blank, standards, and seawater samples, PO_4_^3−^ measurements were conducted by square wave voltammetry with a frequency of 10 Hz, a step potential of 2 mV, and an amplitude of 100 mV. Between measurements, a wash procedure was performed with cyclic voltammetry in a solution of 0.1 M NaOH for 20 scan cycles at a scan rate of 50 mV/s at a potential range of −0.2 to 0.8 V, followed by rinsing of the flow cell with ultrapure water and subsequent discharge to waste. Before and after the measurements, ultrapure water was passed through the electrochemical cell to rinse the electrodes and glass vessel, and then discharged to waste. After the blank, standard, and wash step measurements, the peristaltic pump flow direction was reversed and the solution was returned to the glass vessel. After the seawater measurement, the solution was transferred directly to the waste container. For the blank, standard, and wash step measurements, the peristaltic pump was set to a flow rate of ~5 mL.min^−1^ (60% of the maximum speed (i.e., 48 rpm) for 170 s) in a clockwise direction, and the same step was repeated in a counter-clockwise direction after the measurement. For seawater analysis, the pump was configured at 60% of maximum speed for 155 s, followed by the addition of 50% (*v*/*v*) H_2_SO_4_ for 25 s. Figure S1 shows the graphical user interface for the control of the peristaltic pump, switching valve, and synchronization with the bi-potentiostat with a description of the components.

### 2.6. Field Testing

**Figure 3:**
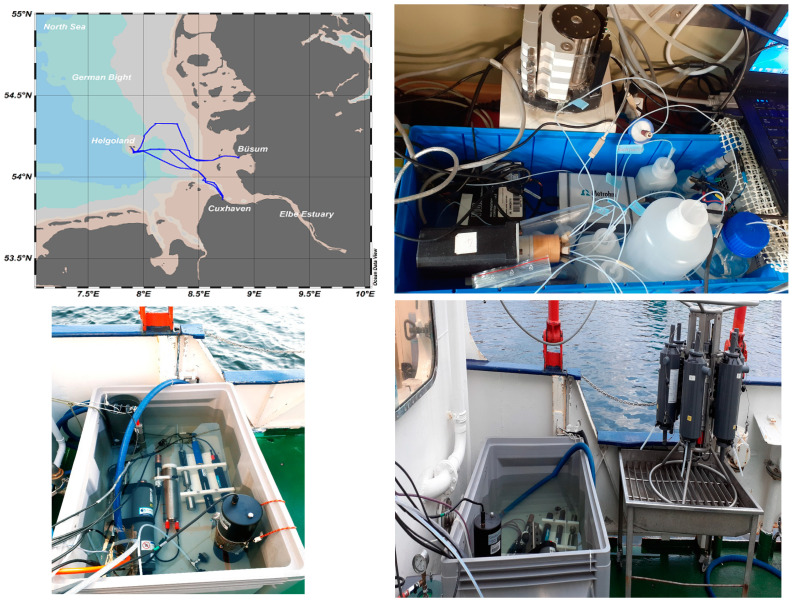
Map showing the track of RV Littorina in German Bight (southeastern North Sea) (**top left**), setup of the FIA-DECD during deployment on the research vessel (**top right**), and a 200 liter water tank with submerged sensors which supplied the FIA-DECD analyzer with surface seawater using a pump placed in the tank (**bottom right and left**).

A field trial was conducted in May–June 2022 on a research vessel in the German Bight of the North Sea, covering the outflow of the Elbe estuary and the region between Cuxhaven, Heligoland, and Büsum. The expedition transect of RV Littorina is shown in Figure 3, top left. The orthophosphate analyzer was set up in the ship’s laboratory (Figure 3, top right) and supplied with a continuous seawater flow. The water was obtained from a 200 L tank which was supplied by surface seawater at a flow rate of 600 L per h (Figure 3, bottom right and left), and also housed several submersible sensors to collect additional hydrographic data. A YSI EXO2 probe was used to monitor salinity, temperature, and dissolved oxygen (DO) at a frequency of 1 min. Sunburst SAMI pH and Trios Opus [29] were used to monitor pH and nitrate (NO_3_^−^) at 15 min and 1 min intervals, respectively. Raw pH and NO_3_^−^ data were corrected for salinity, temperature, and pressure data obtained from the EXO probe. The sample inlet of the FIA-DECD analyzer was fitted with a 0.45 µm syringe filter to remove particles. The analyzer was equipped with a blank solution and three standard solutions (0.2, 1, 2 µM PO_4_^3−^), all prepared in artificial seawater (27 g/L NaCl) (pH 0.8).

Discrete samples for validation of the analyzer performance were collected from the sampling line outlet, filtered through a 0.45 µm syringe filter attached to a 60 mL acid-washed syringe, filled into pre-cleaned 15 mL low-density propylene tubes, and frozen (−20 °C) until analysis at GEOMAR with an air-segmented multi-macronutrient analyzer (QuAAtro, Seal Analytical Ltd.). GPS coordinates (longitudes and latitudes) were obtained from NEMA data from the GEOMAR Littorina vessel and converted to comma-separated value (csv) files.

## 3. Results and Discussion

### 3.1. Dual-Channel Electrochemical PO_4_^3−^ Measurement

In this study, we developed an improved electrochemical analyzer for orthophosphate in seawater through the introduction of a novel dual electrochemical detector cell. The cell houses a working electrode that contains the reagent molybdate in a CPE and acts as a reagent channel, and there is a second working electrode that only contains carbon paste and acts as a reference channel.

The dual electrochemical cells detector uses a bi-potentiostat, where two working electrodes share the same reference and counter electrode. We applied the same potential range over both channels, with the two working electrodes exposed to the same conditions in the same aqueous medium. Previously, we tested the best conditions for the determination of orthophosphate in seawater [22], which was acidified to pH 0.8 to ensure the exclusion of silicic acid interference. Silicic acid is considered the main interference for orthophosphate analysis, as it has the ability to combine with molybdate to form a silicomolybdate complex. A pH range of 0.4–0.9 was recommended to ensure a molybdate/proton ratio of 60–90, thus excluding silicic acid interference [30,31].

**Figure 4:**
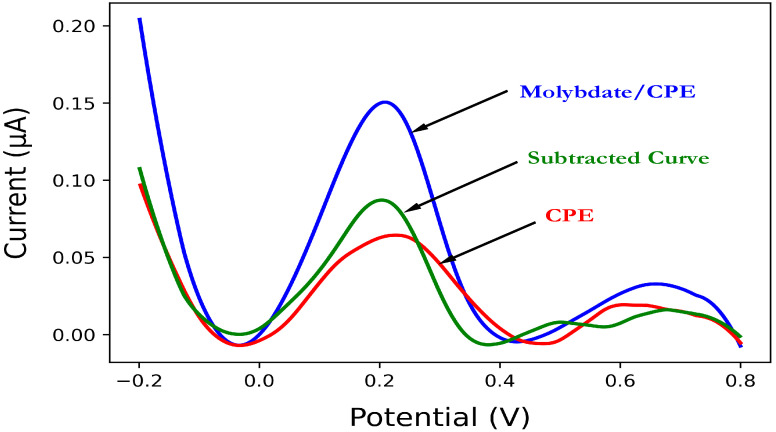
Square wave voltammograms of molybdate/CPE (blue line) and CPE (red line) in a solution of 0.1 µM PO_4_^3−^ in 35 g/L NaCl (pH 0.8) obtained using step potential 2 mV, square amplitude 10 mV, and square frequency 10 mV. The resulting voltammogram after subtraction of the CPE signal from the molybdate/CPE signal is also shown (green line).

The main principle of our method is based on the pre-treatment of the molybdate-modified CPE with cyclic voltammetry in 0.1 M NaOH for 10 cycles to facilitate the formation of molybdate ions. The formed ions readily react with orthophosphate and form the phosphomolybdate complex, which is electrochemically detected with a characteristic peak at ~0.2 V. In the dual electrochemical detection mode, the second working electrode, which does not contain molybdate, is subjected to the same routine, and its voltammogram is subtracted from that of the reagent electrode (molybdate/CPE) to correct for the influence of the matrix interferences (Figure 4).

### 3.2. The Influence of Square Wave Voltammetry Parameters

The most effective way to increase the sensitivity of voltammetric measurements is to perform the analysis in such a way that the contribution of the charging current is reduced [32,33].

This is achieved by replacing the cyclic voltammetry (CV) technique with a continuous potential ramp by square wave voltammetry which employs a staircase potential time modulation with small potential pulses.

**Figure 5:**
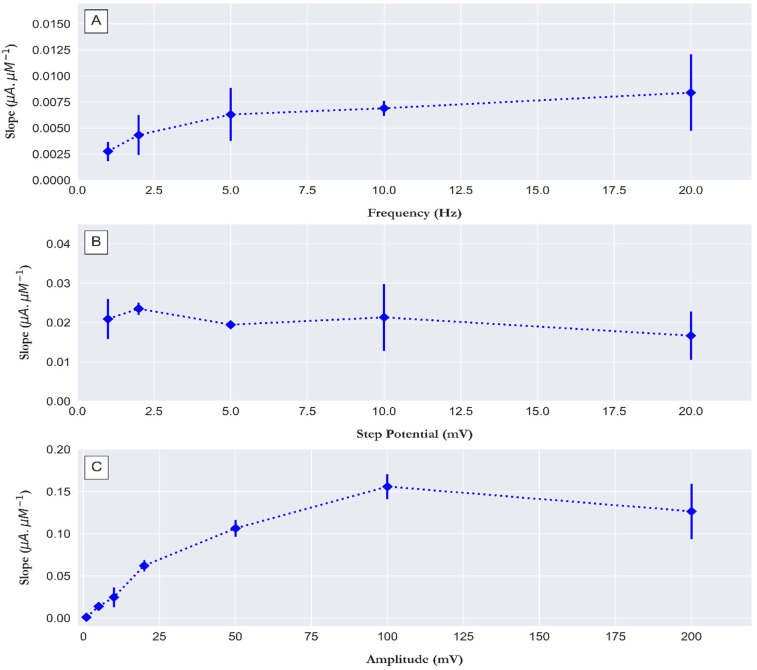
(**A**) The effect of varying square wave frequency from 1 to 20 Hz, (**B**) the effect of varying step potential from 1 to 20 mV, and (**C**) and the effect of square wave amplitude from 1 to 200 mV on the slope of a calibration plot constructed from the corrected square wave voltammogram’s peak current of 0, 0.5, and 1 µM PO_4_^3−^ in 30 g/L NaCl (pH 0.8). Error bar (n = 5).

The reduction in charge current in square wave voltammetry is achieved by measuring the current at the end of each potential step and these techniques are called current-sampled cyclic staircase voltammetry. The main tuning parameters of square wave voltammetry are the sampling increment or step potential (ΔE) and the duration of the step potential (Ʈ). For each potential step, two potential pulses are performed, where the duration of the potential pulses is equal and is denoted as $t_{p}=Ʈ/2$, and the magnitude of each potential pulse is denoted as the square wave amplitude. The duration of the potential step Ʈ is expressed by the frequency (f) in Hz (reciprocal of the second (s^−1^)) $ƒ=\frac{1}{2t_{p}}$, and the sampling rate is expressed by the step potential and frequency as follows υ=ƒΔE [16,34]. Previously, we investigated the effects of the three parameters (i.e., step potential, amplitude, and frequency) on the signal of a PO_4_^3−^ measurement on molybdate/CPE by applying a single electrochemical cell potentiostat. Here, we repeated the determination of the effect of these parameters, as we worked in a dual electrochemical cell detector coupled with a flow injection analysis mode. This flow injection mode is different from the manual batch mode. In flow injection analysis, measurements are performed under hydrodynamic conditions, which differ from the manual batch mode where measurements are performed in a stationary sample solution. We tested the effect of these parameters on the slope with the bi-potentiostat, which can be referred to as the sensitivity of a calibration curve of 0, 0.5, and 1 µM PO_4_^3−^ in 30 g/L NaCl (pH 0.8). Figure 5A shows the effect of varying the frequency from 1 Hz to 20 Hz. The sensitivity increased from 1 Hz with a mean value of 0.0027 µA.µM^−1^ to 10 Hz with a mean value of 0.0069 µA.µM^−1^ (±0.0007), which further increased to a mean value of 0.0084 µA.µM^−1^ with a standard deviation of 0.003 µA.µM^−1^ at 20 Hz. The high standard deviation at 20 Hz could be due to noise which increases when the frequency increases above 10 Hz, as shown in Figure S2, where the voltammograms of 1 µM PO_4_^3−^ at frequencies from 1 Hz to 20 Hz are shown. The step potential mainly controls how many points are sampled over the assigned potential range, and the number of points sampled decreased as the step potential increases from 1 to 20 mV (Figure S3). Figure 5B shows the effect of varying the step potential from 1 to 20 mV on the slope of the calibration. The slope increased from 0.0209 µA.µM^−1^ at 1 mV to 0.0235 µA.µM^−1^ at 2 mV and decreased to 0.01945 µA.µM^−1^ at 5 mV. The amplitude of the square wave controls the width of the curve obtained. Figure S4 shows the voltammograms of 1 µM PO_4_^3−^ at different amplitudes of the square wave from 1 to 200 mV. Figure 5C shows the effect of varying the square wave amplitude from 1 to 200 mV on the slope of the calibration curve, which increases 100-fold from 0.00103 µA.µM^−1^ at 1 mV to 0.106 µA.µM^−1^ at 50 mV, increasing to 0.155 µA.µM^−1^ at 100 mV, and dropping to 0.12 µA.µM^−1^ at 200 mV, which can be attributed to the increased peak width and opening over the potential range from −0.2 to 0.8 V (Figure S4). Overall, the following optimal conditions were chosen for the square wave parameters: 10 Hz frequency, 2 mV step potential, and 100 mV amplitude.

### 3.3. Influence of Salinity Variation

The effect of salinity on the peak signal was studied in detail since salinity can vary strongly in estuaries and coastal waters compared to fresh and open ocean waters. The effect of salinity on electrochemical processes can be explained by the fact that the dominant salt sodium chloride is a strong supporting electrolyte. The effect of salinity was studied by measuring the peak current of a solution of 1 µM PO_4_^3−^ (pH 0.8) at different salinities, for which different amounts of sodium chloride were dissolved in ultrapure water.

The results were plotted against the peak signal of molybdate/CPE before and after applying the correction with CPE to investigate whether this correction can be applied to compensate for the effect of salinity variation. Figure S5 shows the peak current of the square wave voltammogram for molybdate/CPE (uncorrected) in ultrapure water S = 0 (0.0012 µA), with the peak current increasing with increasing salinity S = 7 (0.09 µA). A slight increase in the peak current with increasing salinity to S = 14 (0.113 µA) reaches a steady state with increasing salinity to S = 21 (0.116 µA). A high rise was obtained with increasing salinity to S = 28 (0.149 µA). In the corrected voltammogram after subtracting the CPE signal from the molybdate/CPE signal, the peak current in ultrapure water was S = 0 (0.00016 µA), which increased with increasing salinity S = 7 (0.038 µA). An increase in peak current was obtained with increasing salinity up to S = 14 (0.102 µA), and a steady state was obtained at S = 21 (0.108 µA). A sharp increase in peak current was obtained by increasing the salinity to S = 28 (0.136 µA). A comparison between the two values showed that the values obtained from the corrected voltammogram were underestimated by 150% (S = 7) compared to the uncorrected voltammogram. The underestimation decreased with increasing salinity to S = 14 (10.9%), S = 21 (7.6%), and S = 28 (9.4%). Overall, the correction was not adequate to compensate for the variation in salinity. This could be because with increasing sodium chloride concentration, high concentrations of phosphomolybdate complexes can form at the molybdate/CPE. This is related to the fact that most of the oxidation, which refers here to the coupling between PO_4_^3−^ and molybdate, is mediated by the electrogenerated chloro-species [35]. This could not occur at the CPE as it does not contain molybdate. This makes the correction (i.e., subtracting of the CPE signal from the molybdate/CPE signal) not useful for salinity variation compensation. Therefore, the recommendation to prepare calibration standards before field deployment with a salinity close to that of the waters under investigation persists.

### 3.4. Interferences between Surfactant and Humic Acid

**Figure 6:**
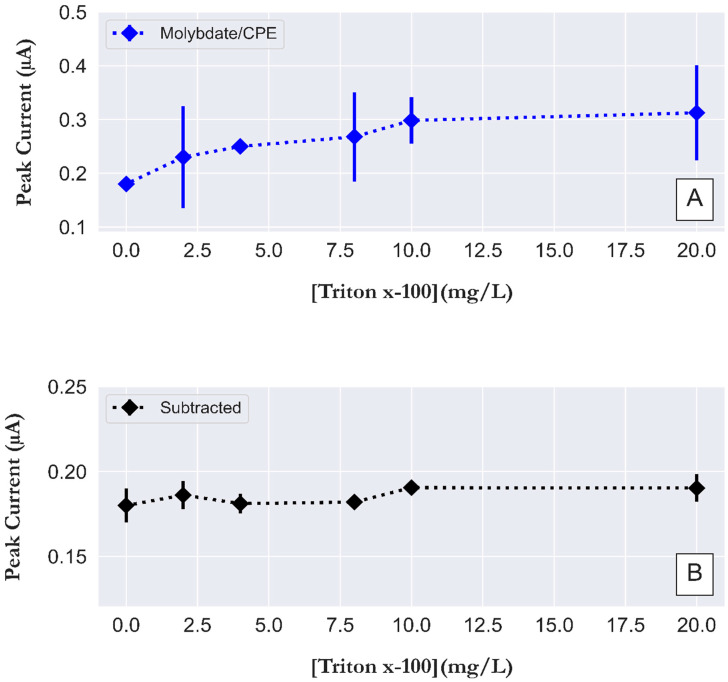
Interference effect from non-ionic surfactant (Triton x-100) on the square wave voltammograms peak current (µA) at (**A**) molybdate/CPE and (**B**) the resulting voltammogram obtained after subtraction of CPE signal from the molybdate/CPE of 1 µM PO_4_^3−^ in 30 g/L NaCl, pH 0.8. The parameters for square wave voltammetry were a step potential of 2 mV, an amplitude of 50 mV, and a frequency of 10 Hz. Error bar (n = 5).

A surfactant can alter the properties of the interface between the electrode and sample solution, affecting electrochemical processes and thus the peak signal [36]. A surfactant is a substance with hydrophilic moieties on its head and hydrophobic moieties on its tail. One of the non-ionic surfactants is Triton x-100, which is classified as a strongly hydrophobic surfactant with about 95 ethoxy groups [37]. The influence of Triton x-100 on our peak currents was tested by a series of concentrations of Triton x-100 in a solution of 1 µM PO_4_^3−^ (pH 0.8), and the peak current of molybdate/CPE and the corrected voltammogram were measured and compared to determine the improvements obtained by the application of our bi-potentiostat method. Figure 6A shows the peak current of the voltammogram for molybdate/CPE (uncorrected). The results indicate that the addition of 2 mg/L Triton x-100 resulted in a 27% increase in peak current compared to a surfactant-free standard solution of 1 µM PO_4_^3−^ with a relative standard deviation (RSD) of 41%. For a solution of 10 mg/L Triton x-100, the increased peak current resulted in an overestimation of PO_4_^3−^ by ~65% with an RSD of 14%. For 20 mg/L Triton x-100, an increase in peak current resulted in an overestimation of PO_4_^3−^ of ~73% with an RSD of 28%. The peak current values obtained from the corrected voltammogram (Figure 6B) showed a slight increase in peak current with the addition of Triton x-100 compared to that of the surfactant-free solution of 1 µM PO_4_^3−^. The increased peak current after the addition of Triton x-100 from 2 mg/L to 20 mg/L leads to a maximum overestimation of PO_4_^3−^ of ~6%, with RSD values in the range of 5%. The RSD values are lower than the value suggested by Gibbons et al. [38] as acceptable for environmental analyses and indicate good precision of the analyzer after our correction for the presence of Triton x-100. The results show that our bi-potentiostat method is able to correct for interference caused by surfactants (Triton x-100), which may be present in surface seawaters in the concentration range of 0.1 to 2 ppm [39].

Humic acid (HA) is classified as a surface-active compound that alters the electrochemical process and thus the voltammetric signal, as HA affects the interface of the electrode surface like a surfactant [40]. Figure S6 indicates that at 1 mg/L HA suppresses the voltammetric signal of 1 µM PO_4_^3−^, and the signal was tested in solutions with concentrations up to 20 mg/L.

The results showed a strong effect of HA on the signal, which is possibly due to the decreasing functional activity of the carbon paste surface by adsorption of HA. This is likely related to a high adsorption capacity of HA on CPEs [41].

### 3.5. Analytical Performance

**Figure 7:**
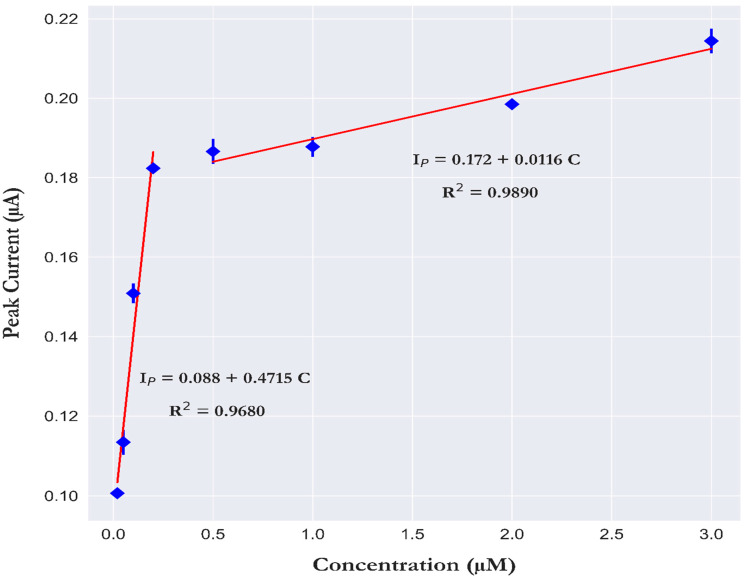
Calibration plots for two concentration ranges. Low range (0.02, 0.05, 0.1, and 0.2 µM PO_4_^3−^) and high range (0.5, 1, 2, and 3 µM PO_4_^3−^) in 30 g/L NaCl (pH 0.8), a step potential of 2 mV, an amplitude of 50 mV and a frequency of 10 Hz. Error bar (±σ) (n = 5).

Measurements were performed at a time interval of 30 min with a wash step between the measurements (cyclic voltammetry in 0.1 M NaOH, scan rate 0.1 Vs^−1^ for 20 scan cycles). The square wave voltammetry of the molybdate/CPE was corrected by the square wave voltammetry of the CPE. Figure 7 indicates that two ranges of linear calibration plots were obtained, with a first linear range for concentrations of 0.02, 0.05, 0.1, and 0.2 µM PO_4_^3−^, with an analytical sensitivity of 0.4715 µA.µM^−1^ and a detection correlation (R^2^) of 0.968. The second linear range was for concentrations of 0.5, 1, 2, and 3 µM PO_4_^3−^, with an analytical sensitivity of 0.0116 µA.µM^−1^ and a detection correlation (R^2^) of 0.989.

The division of the two discrete linear regions was also observed in our previous work [22] and explained by the adsorption of phosphomolybdate complex layers on the working electrode surface at high PO_4_^3−^ concentrations. Calibration standards should hence be prepared with concentrations close to the level of PO_4_^3−^ expected in the study region.

Similar behavior was noticed for the colorimetric determination of PO_4_^3−^ based on the Molybdate Blue method. This is illustrated by the fact that there is no report of a single instrument suitable for measuring PO_4_^3−^ in a concentration range from nanomolar to micromolar levels [42,43]. The limit of detection (LOD) of our method was calculated as three times the standard deviation of the blank measurements [44,45] and was 0.014 µM PO_4_^3−^.

As part of the evaluation of the analytical performance of our method, the accuracy of FIA-DCED was tested. For this purpose, five replicate measurements of certified reference material (CRM) (Kanso Co., Ltd., Osaka, Japan) were analyzed. The batches CRM CG, CI, and CH were analyzed with the following assigned values for PO_4_^3−^ concentration: 1.70 ± 0.0011 µM, 0.948 ± 0.002 µM and 1.172 ± 0.0016 µM, respectively. The mean values of the measured values were as follows: 1.552 ± 0.125 µM, 1.04 ± 0.0917 µM, and 1.188 ± 0.0599 for CRM CG, CI, and CH, respectively. Systematic error (bias) was also determined using the two-tailed paired *t*-test at a degree of freedom (*df*) of 4 and a significance level of 1%. No bias was detected in the measured values: (*t*-value = 2.36, t_critical_ value = 4.604, *p* ˃ 0.01) for CRM CG. While for CRM CI (*t*-value = −1.75, t_critical_ value = 4.604, *p* ˃ 0.01), and for CRM CH (*t*-value = −0.526, t_critical_ value = 4.604, *p* ˃ 0.01). Here, no significant difference was found between the measured and assigned values. This demonstrates the ability of our analyzer to realistically measure PO_4_^3−^ in seawater.

### 3.6. Field Deployment

Validation of the new method took place during a research cruise with the vessel Littorina in the German Bight (southern North Sea). Field tests were conducted in the outflow of the Elbe estuary in the region of the North Sea between Heligoland (54.180327 N, 7.888944 E) and Cuxhaven (53.859336 N, 8.687906 E), and between Heligoland and Büsum (54.134622 N, 8.858591 E). The main sources of nutrients to the shallow German Bight come from the west, including the English Channel, and via river discharges (e.g., Elbe, Rhine, Maas, Weser, Ems) [46], but also from atmospheric deposition [47].

One of the main influences on orthophosphate levels in the North Sea around Heliogland is river discharge. This clearly appeared in other studies in the same region where orthophosphate exhibited significant correlations with salinity [48,49]. Typically, orthophosphate concentrations are related to the primary production and decomposition of organic matter. In the North Sea, orthophosphate exhibits a seasonal cycle, with enhanced concentrations in late summer and autumn with an average of 1 µM, and low levels in April and May with an average concentration of 0.2 µM [50]. In early summer through October/November, a gradual increase in orthophosphate concentration is observed. This is due to the release of orthophosphate by the decomposition of organic matter, which then exceeds the rate of uptake by phytoplankton [51].

**Figure 8:**
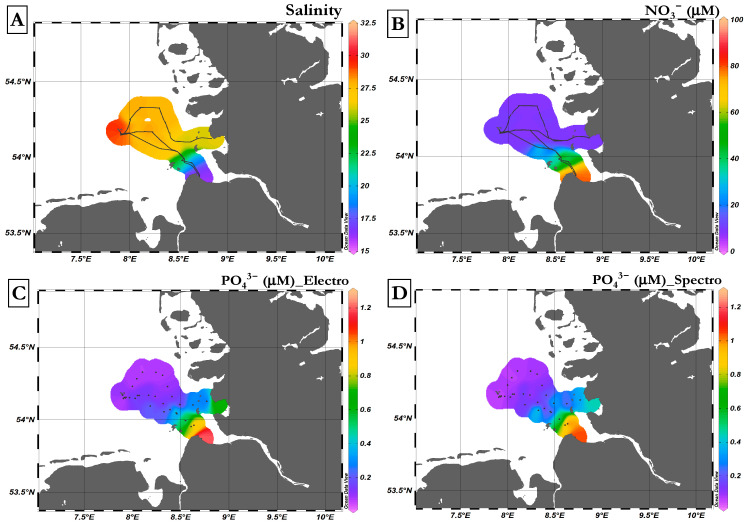
Contour plots of variables in the study region. (**A**) The distribution of surface salinity, (**B**) the distribution of NO_3_^−^ concentration determined with the Trios sensor OPUS, (**C**) the distribution of PO_4_^3−^ concentration determined in discrete samples collected and analyzed with an electrochemical analyzer (FIA-DECD), and (**D**) the distribution of PO_4_^3−^ concentration determined in discretely collected samples and analyzed at GEOMAR using a spectrophotometric analyzer. Maps plotted via ODV 5.3.0 [52].

Figure 8A shows the distribution of salinity obtained with the CTD EXO probe during the Littorina cruise, with a minimum of 15.5 near Cuxhaven and a maximum value of 30.26 near Heligoland. The data obtained show that salinity increased gradually as the vessel sailed away from the coast of Cuxhaven, Büsum, and the mouth of the Elbe River, toward the island of Heligoland. The distribution of dissolved oxygen (DO) (Figure S8A) showed concentrations of 9.5–10 mg/L near Cuxhaven, and a decrease toward Heligoland (8.5–9 mg/L), including a clear minimum (<8–7 mg/L). The pH values recorded by the Sunburst SAMI sensor at a 15 min time intervals (see Figure S8B) were consistent with the salinity data, with the lowest values (pH 8.106) measured near Cuxhaven, and an increase toward Heligoland (8.2–8.4). The distribution of nitrate (Trios OPUS sensor) mirrored salinity (Figure 8B) with maximum values (˃80 µM–100 µM) near Cuxhaven and a decrease (<20 µM–5 µM) toward Heligoland. Similar patterns were obtained for ∑(NO_3_^−^ + NO_2_^−^) from the discretely collected samples that were analyzed at GEOMAR (Figure S8C). A maximum concentration of 55.9 µM was reached near Cuxhaven, and levels gradually decreased and remained in the low range (<10 µM–~1 µM) toward Heligoland Island and also toward Büsum. A total of 36 measurements were conducted at sea for PO_4_^3−^ with our analyzer (FIA-DECD) at a time interval of 30-min. The distribution of PO_4_^3−^ (Figure 8C) showed a similar distribution as nitrate, with peak values near Cuxhaven (~1 µM), gradually decreasing toward Heligoland and slightly increasing toward Büsum. A similar distribution was seen for PO_4_^3−^ from discretely collected samples with analysis using the standard spectrophotometric technique (34 discrete samples; Figure 8D). The distribution of H_4_SiO_4_ (see Figure S8D) coincided with the PO_4_^3−^ distribution, with maximum values (~15 µM) near Cuxhaven and a decreasing trend in an offshore direction with low concentrations (1–4 µM) near Heligoland and also near the coast toward Büsum.

**Figure 9:**
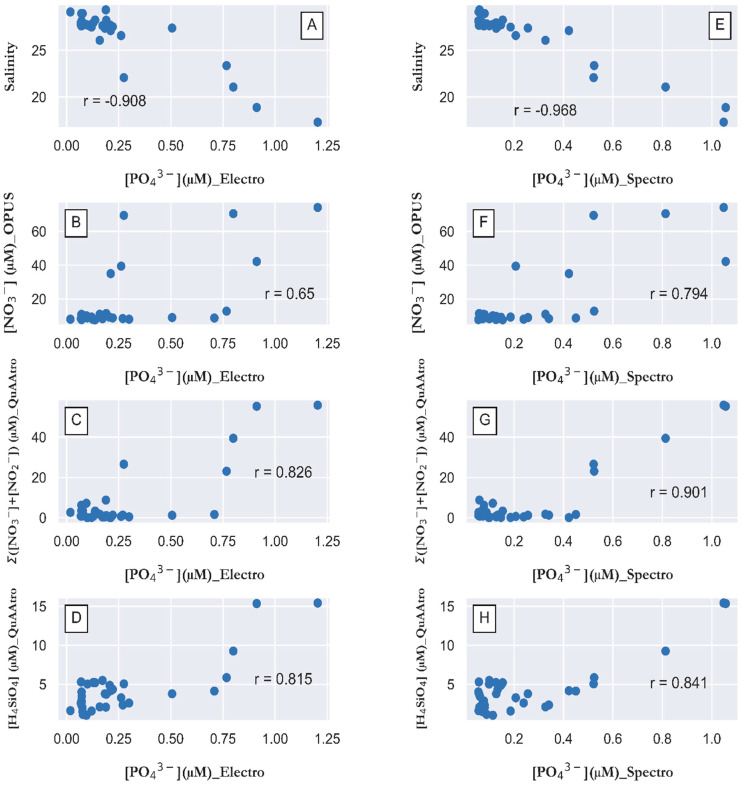
Property–property plots for electrochemically measured PO_4_^3−^ concentration in µM compared with (**A**) salinity (Pearson’s r = −0.908, n = 31), (**B**) NO_3_^−^ concentration in µM determined from a Trios OPUS sensor (Pearson’s r = 0.65, n = 34), (**C**) Ʃ(NO_3_^−^ + NO_2_^−^) concentration in µM analyzed in discrete samples with a QuAAtro analyzer (Pearson’s r = 0.826, n = 34), and (**D**) H_4_SiO_4_ concentration in discrete samples analyzed with a QuAAtro spectrophotometric analyzer (Pearson’s r = 0.815, n = 34), and for PO_4_^3−^ concentration in µM measured spectrophotometrically with the QuAAtro air segment analyzer versus (**E**) salinity (Pearson’s r = −0.968, n = 31), (**F**) NO_3_^−^ concentration in µM from a Trios OPUS sensor (Pearson’s r = 0.794, n = 34), (**G**) ∑(NO_3_^−^ + NO_2_^−^) concentration in µM analyzed in discrete samples with the QuAAtro analyzer (Pearson’s r = 0.901, n = 34), and (**H**) H_4_SiO_4_ concentration in discrete samples analyzed with the QuAAtro spectrophotometric analyzer (Pearson’s r = 0.841, n = 34).

The most important factor in the distribution of macronutrients in the studied area is the discharge of river water and the mixing of the nutrient-enriched river water with North Sea waters containing lower nutrient concentrations. This is clearly shown in Figure 9, which summarizes all significant correlations. A strong negative significant correlation was found between salinity and PO_4_^3−^ concentrations measured by FIA-DCED (Figure 9A), with a Pearson correlation coefficient of −0.908. Almost the same behavior was found for PO_4_^3−^ concentrations measured spectrophotometrically (Figure 9E), with a correlation coefficient of −0.968. Therefore, the distribution shown for PO_4_^3−^ should naturally be very close to the distributions of the other macronutrients, which are nitrate (NO_3_^−^) and silicic acid (H_4_SiO_4_), which is very important for silicifying phytoplankton species, such as diatoms. This behavior was evident when comparing the real-time monitoring data for NO_3_^−^ concentration collected with the Trios sensor OPUS with the measured PO_4_^3−^ concentration. Here, a correlation coefficient of 0.65 was obtained for the electrochemically measured values (Figure 9B) and 0.794 for the spectrophotometrically measured values (Figure 9F). The concentrations of nitrate plus nitrite (∑(NO_3_^−^ + NO_2_^−^) and H_4_SiO_4_ in the discrete samples analyzed by standard spectrophotometric methods correlate with PO_4_^3−^ concentrations. Strong positive correlation coefficients of 0.826 and 0.815 were obtained for the electrochemically measured PO_4_^3−^ concentrations with the ∑(NO_3_^−^ + NO_2_^−^) (Figure 9C) and H_4_SiO_4_ concentrations (Figure 9D), respectively. Slightly increased correlation coefficients were obtained between spectrophotometrically measured PO_4_^3−^ concentrations, with values of 0.901 and 0.841 with ∑(NO_3_^−^ + NO_2_^−^) (Figure 9G) and H_4_SiO_4_ concentrations (Figure 9H), respectively.

Overall, the results showed that FIA-DCED is able to provide high-quality data on PO_4_^3−^ concentrations that contribute to a better understanding of the distribution in coastal marine systems. This was evidenced by the relationships with other parameters, such as salinity and other macronutrients. This showed that the FIA-DCED is very well-suited for accurate on-site determination of PO_4_^3−^.

The waters in the Kiel Canal are characterized by a high concentration of suspended solids and thus turbidity and low salinity. The mean salinity and turbidity values obtained with the EXO probe were 4.92 ± 1.38 and 137.71 ± 36.17 FNU, respectively (from 53.884905 N 9.139106667 E to 54.01754 N, 9.297675 E). The canal was characterized by high organic matter content [53]. Three points were sampled in the Kiel Canal, as shown in Figure S7, although the presence of organic matter in the form of humic acid greatly affected the voltammogram of the electrode (Section 3.5). The voltammogram of the three points showed no noise, demonstrating the suitability of the method for the determination of orthophosphate in environmental samples with high concentrations of organic matter.

Bio-fouling is not an important issue for our instrument, as the acidic reagent solution and the regular rinsing with NaOH solution will prevent growth inside the sensor. In future applications, we will also include a syringe filter with a small pore size (e.g., 1 μm) to prevent entry into the analyzer of particles and thereby the possibility of internal fouling.

The electrochemical method was validated against the standard laboratory autoanalyzer. The concentrations obtained by the new bi-potentiostat analyzer averaged 0.26 µM, with minimum and maximum values of 0.018 µM and 1.2 µM, respectively. Concentrations obtained by the colorimetric analyzer averaged 0.24 µM, with minimum and maximum values of 0.057 µM and 1.056 µM, respectively. Considering all data points, the mean accumulation level is 108% (evaluated as the average of the recovery values). The poor recoveries (~30%) were obtained at values below 0.06 µM, which could be due to the deviation of the linearity of the calibration curve when measuring values in the nanomolar range (20–50 nM) and values in the micromolar range (0.2–3 µM), as shown in Section 3.5.

**Figure 10:**
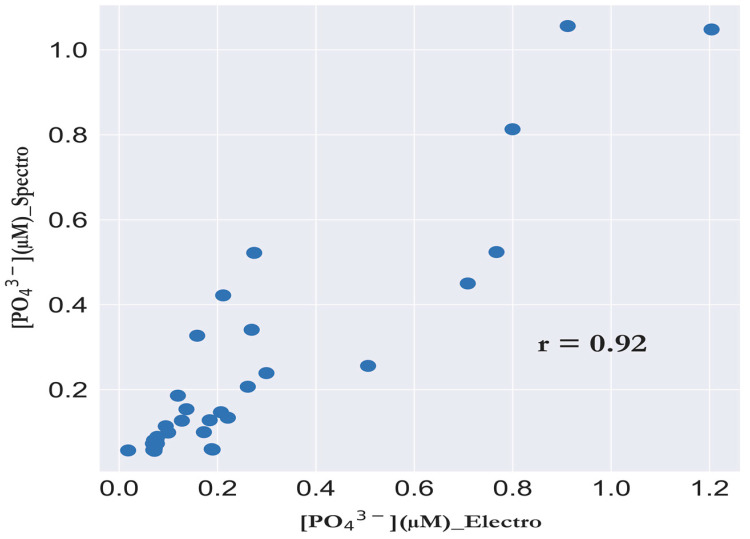
Scatter plot for *on-site* PO_4_^3−^ concentrations (µM) measured electrochemically via FIA-DECD versus PO_4_^3−^ concentrations (µM) measured in discrete samples collected and analyzed via a reference colorimetric laboratory-based analyzer. Pearson’s R = 0.917.

The correlation plot between PO_4_^3−^ concentrations obtained using the new analyzer and standard laboratory technique (Figure 10) showed a good correlation coefficient (R^2^) of 0.917 (n = 34), and also the paired *t*-test shows that there is no significant difference between the means at 1% level (*p*-value = 0.40597, *df* = 33) with the null hypothesis (mean (*on-site* data) = mean (discrete samples)) and the alternative hypothesis (mean (*on-site* data) − mean (discrete samples) < > 0).

## 4. Conclusions and Suggestions for Future Work

Here, we presented the application of a bi-potentiostat in an autonomous analyzer for the electrochemical determination of PO_4_^3−^. The work forms an application of our recently published method for the *on-site* determination of PO_4_^3−^ in seawater using molybdate/CPE after pre-treatment with NaOH. Two working electrodes were used, molybdate/CPE and CPE, to correct matrix interference and increase the reliability of the method. The integration of the flow injection analyzer (peristaltic pump and switching valve) in conjunction with data processing using Python software allowed full automation of the analyzer. The method, with an analysis frequency of 30 min, exhibited a wide detection range of 0.2–3 µM with a LOD of 0.14 µM. The method was validated for *on-site* determination of PO_4_^3−^ in the coastal waters of the North Sea on the RV Littorina with other sensors deployed in addition to our analyzer. The analyzer performed well compared to the laboratory colorimetric reference methods. Using the analyzer, the spatial distribution of PO_4_^3−^ in the German Bight in the discharge plume of the Elbe estuary could be determined with patterns that correlated highly with other ancillary hydrographic data. To further improve the method, an electrochemical flow cell with a low flow rate could be used both to reduce reagent consumption and to shorten measurement times. The use of a syringe pump instead of a peristaltic pump could help to reduce reagent consumption since syringe pumps are able to deliver smaller solution volumes (10 µL).

## Data Availability

Data is contained within the article and the Supplementary Materials.

## Supplementary Materials

The following are available online at https://www.mdpi.com/article/10.3390/s23042123/s1, Figure S1: graphical user interface (GUI) of the ‘Electrochemical FIA.exe’ to program switching valve, peristaltic pump, and synchronization with the Metrohm µStat 400 Bi-potentiostat. Figure S2: Square wave voltammograms of 0.5 µM PO_4_^3−^ in 30 g/L NaCl (pH 0.8) (corrected voltammogram) at a step potential of 1 mV, square wave amplitude 25 mV at frequencies 1 Hz, 2 Hz, 5 Hz, 10 Hz, and 20 Hz. Figure S3: Square wave voltammograms of 0.5 µM PO_4_^3−^ in 30 g/L NaCl (pH 0.8) (corrected voltammogram) at a frequency of 10 Hz, amplitude 25 mV at a step potential of 1 mV, 2 mV, 5 mV, 10 mV, and 20 mV. Figure S4: Square wave voltammograms of 0.5 µM PO_4_^3−^ in 30 g/L NaCl (pH 0.8) (corrected voltammogram) at a step potential of 1 mV and a frequency of 10 Hz at square wave amplitudes of 1 mV, 5 mV, 10 mV, 20 mV, 50 mV, 100 mV, and 200 mV. Figure S5: Effect of the variation of the salinity (0, 7, 14, 21, and 28) on the peak current of 1 µM PO_4_^3−^ (pH 0.8) where the peak current of molybdate/CPE is shown as black circles and the peak current of corrected voltammogram is shown as blue circles. Error bar (n = 5). Figure S6: Square wave voltammograms of 1 µM PO_4_^3−^ (30 g/L NaCl) pH 0.8 on molybdate/CPE (blue line) and CPE (orange line) in the presence of 1, 2, 5, 10, and 20 mg/L HA. Step potential 2 mV, frequency 10 Hz and amplitude of square wave 100 mV. Figure S7: Location of three on-site data points taken at the Kiel Canal with square wave voltammograms at molybdate/CPE (blue line) and CPE (red line). The left inset shows the turbidity of the water entering the tank. Figure S8: Overviews of (A) the distribution of surface dissolved oxygen (DO) in mg/L obtained from the EXO sonde sensor, (B) the distribution of pH obtained from sunburst SAMIpH and corrected for CTD salinity and temperature, (C) the distribution of ∑(NO_3_^−^ + NO_2_^−^) in µM (bottom left panel), and (D) the distribution of H_4_SiO_4_ in µM for the discrete samples collected from underway water supply and analyzed via a QuAAtro air-segmented analyzer.

## References

[1] Van Cappellen P., Ingall E.D. (1996). Redox stabilization of the atmosphere and oceans by phosphorus-limited marine productivity. Science.

[2] McDowell R.W., Hamilton D.P. (2013). Nutrients and eutrophication: Introduction. Mar. Freshw. Res..

[3] Canfield D., Wollast R., Mackenzie F., Chou L. (1993). C, N, P, S Global Biogeochemical Cycles and Modeling of Global Change. Interactions of C, N, P and S Biogeochemical Cycles and Global Change.

[4] Puchades R., Maquieira A., Atienza J., Herrero M. (1990). State of the art in on-line techniques coupled to flow injection analysis FIA/on-line-a critical review. J. Autom. Chem..

[5] Murphy J., Riley J.P. (1962). A modified single solution method for the determination of phosphate in natural waters. Anal. Chim. Acta.

[6] Heidari-Bafroui H., Charbaji A., Anagnostopoulos C., Faghri M. (2021). A Colorimetric Dip Strip Assay for Detection of Low Concentrations of Phosphate in Seawater. Sensors.

[7] Kitson R.E., Mellon M.G. (1944). Colorimetric Determination of Phosphorus as Molybdivanadophosphoric Acid. Ind. Eng. Chem. Anal. Ed..

[8] Willsky G.R., White D.A., McCabe B.C. (1984). Metabolism of added orthovanadate to vanadyl and high-molecular-weight vanadates by Saccharomyces cerevisiae. J. Biol. Chem..

[9] Snazelle T.T. (2018). Laboratory Evaluation of the Sea-Bird Scientific HydroCycle-PO4 Phosphate Sensor.

[10] Beaton A.D., Schaap A.M., Pascal R., Hanz R., Martincic U., Cardwell C.L., Morris A., Clinton-Bailey G., Saw K., Hartman S.E. (2022). Lab-on-chip for in situ analysis of nutrients in the deep sea. ACS Sens..

[11] Bodini S., Sanfilippo L., Savino E., Moscetta P. (2015). In Automated micro loop flow reactor technology to measure nutrients in coastal water: State of the art and field application. Proceedings of the OCEANS 2015—Genova.

[12] Bohlen C., Liebman M. (2019). Quality Assurance Project Plan for Field Deployment of an Autonomous Nutrient Monitor in Casco Bay.

[13] Grand M.M., Clinton-Bailey G.S., Beaton A.D., Schaap A.M., Johengen T.H., Tamburri M.N., Connelly D.P., Mowlem M.C., Achterberg E.P. (2017). A Lab-On-Chip Phosphate Analyzer for Long-term In Situ Monitoring at Fixed Observatories: Optimization and Performance Evaluation in Estuarine and Oligotrophic Coastal Waters. Front. Mar. Sci..

[14] Green Eyes, LLC. http://gescience.com/wp-content/uploads/2017/02/Green-Eyes-Data-Processing-Guide-1.pdf.

[15] Altahan M.F., Esposito M., Achterberg E.P. (2022). Improvement of On-Site Sensor for Simultaneous Determination of Phosphate, Silicic Acid, Nitrate plus Nitrite in Seawater. Sensors.

[16] Mirceski V., Gulaboski R. (2014). Recent achievements in square-wave voltammetry (a review). Maced. J. Chem. Chem. Eng..

[17] Kolliopoulos A.V., Kampouris D.K., Banks C.E. (2015). Rapid and Portable Electrochemical Quantification of Phosphorus. Anal. Chem..

[18] Jońca J., Fernández V.L., Thouron D., Paulmier A., Graco M., Garçon V. (2011). Phosphate determination in seawater: Toward an autonomous electrochemical method. Talanta.

[19] Barus C., Romanytsia I., Striebig N., Garçon V. (2016). Toward an in situ phosphate sensor in seawater using Square Wave Voltammetry. Talanta.

[20] Jońca J., Giraud W., Barus C., Comtat M., Striebig N., Thouron D., Garçon V. (2013). Reagentless and silicate interference free electrochemical phosphate determination in seawater. Electrochim. Acta.

[21] Cinti S., Talarico D., Palleschi G., Moscone D., Arduini F. (2016). Novel reagentless paper-based screen-printed electrochemical sensor to detect phosphate. Anal. Chim. Acta.

[22] Altahan M.F., Achterberg E.P., Ali A.G., Abdel-Azzem M. (2021). NaOH Pretreated Molybdate-Carbon Paste Electrode for the Determination of Phosphate in Seawater by Square Wave Voltammetry with Impedimetric Evaluation. J. Electrochem. Soc..

[23] Masud J., Liyanage W.P.R., Cao X., Saxena A., Nath M. (2018). Copper Selenides as High-Efficiency Electrocatalysts for Oxygen Evolution Reaction. ACS Appl. Energy Mater..

[24] Hansen E.H., Ruzicka J., Chocholous P. Advances in Flow Injection Analysis. https://www.flowinjectiontutorial.com/index.html.

[25] Thompson N.L. (2018). Total Differential Capacity Plot Analysis Using Data Science Methods. Ph.D. Thesis.

[26] Virtanen P., Gommers R., Oliphant T.E., Haberland M., Reddy T., Cournapeau D., Burovski E., Peterson P., Weckesser W., Bright J. (2020). SciPy 1.0: Fundamental algorithms for scientific computing in Python. Nat. Methods.

[27] Virtanen P., Gommers R., Burovski E., Oliphant T.E., Cournapeau D., Weckesser W., Peterson P., van der Walt S., Mayorov N., Wilson J. (2018). Scipy/Scipy: Scipy 1.1. 0Rc1.

[28] Seabold S., Perktold J. Statsmodels: Econometric and statistical modeling with python. Proceedings of the 9th Python in Science Conference.

[29] Nehir M., Esposito M., Begler C., Frank C., Zielinski O., Achterberg E.P. (2021). Improved calibration and data processing procedures of OPUS optical sensor for high-resolution in situ monitoring of nitrate in seawater. Front. Mar. Sci..

[30] Directive W.F. (2000). Water Framework Directive. J. Ref. OJL.

[31] Jońca J., Comtat M., Garçon V. (2013). In Situ Phosphate Monitoring in Seawater: Today and Tomorrow. Smart Sens. Real-Time Water Qual. Monit..

[32] Murray A.R., Bard I.A. (1984). Electroanalytical Chemistry.

[33] Mirceski V., Komorsky-Lovric S., Lovric M. (2007). Square-Wave Voltammetry: Theory and Application.

[34] Mirceski V., Gulaboski R., Lovric M., Bogeski I., Kappl R., Hoth M. (2013). Square-wave voltammetry: A review on the recent progress. Electroanalysis.

[35] Saxena P., Ruparelia J. (2019). Influence of supporting electrolytes on electrochemical treatability of Reactive Black 5 using dimensionally stable anode. J. Inst. Eng. Ser. A.

[36] Shankar S.S., Swamy B.K., Chandrashekar B. (2012). Electrochemical selective determination of dopamine at TX-100 modified carbon paste electrode: A voltammetric study. J. Mol. Liq..

[37] Kowalcze M., Jakubowska M. (2018). Voltammetric determination of thujone in herbal matrices in the presence of Triton X-100. Anal. Biochem..

[38] Gibbons R.D., Coleman D.E. (2001). Statistical Methods for Detection and Quantification of Environmental Contamination.

[39] Cosović B., Vojvodić V. (1982). The application of ac polarography to the determination of surface-active substances in seawater 1. Limnol. Oceanogr..

[40] Piech R., Baś B., Kubiak W.W. (2008). The cyclic renewable mercury film silver based electrode for determination of molybdenum (VI) traces using adsorptive stripping voltammetry. Talanta.

[41] Zghal S., Jedidi I., Cretin M., Cerneaux S., Abdelmouleh M. (2020). One-step synthesis of highly porous carbon graphite/carbon nanotubes composite by in-situ growth of carbon nanotubes for the removal of humic acid and copper (II) from wastewater. Diam. Relat. Mater..

[42] Ma J., Adornato L., Byrne R.H., Yuan D. (2014). Determination of nanomolar levels of nutrients in seawater. TrAC Trends Anal. Chem..

[43] Deng Y., Li P., Fang T., Jiang Y., Chen J., Chen N., Yuan D., Ma J. (2020). Automated determination of dissolved reactive phosphorus at nanomolar to micromolar levels in natural waters using a portable flow analyzer. Anal. Chem..

[44] Long G.L., Winefordner J.D. (1983). Limit of detection. A closer look at the IUPAC definition. Anal. Chem..

[45] Belter M., Sajnóg A., Barałkiewicz D. (2014). Over a century of detection and quantification capabilities in analytical chemistry—Historical overview and trends. Talanta.

[46] Los F., Troost T., Van Beek J. (2014). Finding the optimal reduction to meet all targets—Applying Linear Programming with a nutrient tracer model of the North Sea. J. Mar. Syst..

[47] Salomons W., Bayne B.L., Duursma E.K., Förstner U. (2012). Pollution of the North Sea: An Assessment.

[48] Raabe T., Wiltshire K.H. (2009). Quality control and analyses of the long-term nutrient data from Helgoland Roads, North Sea. J. Sea Res..

[49] Shchekinova E., Kong S.-M., Boersma M., Wiltshire K.H. (2017). Variations of annual turnover cycles for nutrients in the North Sea, German bight nutrients turnover cycles in the North Sea. Oceanogr. Fish. Open Access J..

[50] Grunwald M., Dellwig O., Kohlmeier C., Kowalski N., Beck M., Badewien T.H., Kotzur S., Liebezeit G., Brumsack H.-J. (2010). Nutrient dynamics in a back barrier tidal basin of the Southern North Sea: Time-series, model simulations, and budget estimates. J. Sea Res..

[51] Van Beusekom J.E., Loebl M., Martens P. (2009). Distant riverine nutrient supply and local temperature drive the long-term phytoplankton development in a temperate coastal basin. J. Sea Res..

[52] Schlitzer R. Ocean Data View, ODV 5.2.1. https://odv.awi.de/.

[53] Schubert B., Krebs F., Bergmann H. (2000). In Federal regulations for the disposal of dredged material in German coastal areas–experiences with chemical and biological criteria. Workshop Report.

