# Supplementary material for: The Use of Bi-Potentiostat as a Simple and Accurate Electrochemical Approach for the Determination of Orthophosphate in Seawater
Source: Sensors (Basel). 2023 Feb 13;23(4):2123. doi: 10.3390/s23042123 (PMC9959667; doi:10.3390/s23042123)
Supplement: Supplementary file 1 [file sensors-23-02123-s001.zip › sensors-2160398-supplementary.pdf]

Use of bi-potentiostat as a simple and accurate electrochemical approach for the determination of orthophosphate in seawater

Mahmoud Fatehy Altahan[1,2], Mario Esposito[1], Boie Bogner[1], Eric P. Achterberg[1]

[1] Chemical Oceanography Department, GEOMAR Helmholtz Centre for Ocean Research, Kiel 24148, Germany.

[2] Central Laboratory for Environmental Quality Monitoring, National Water Research Centre, El-Qanater El- Khairia 13621, Egypt.

Supplementary information

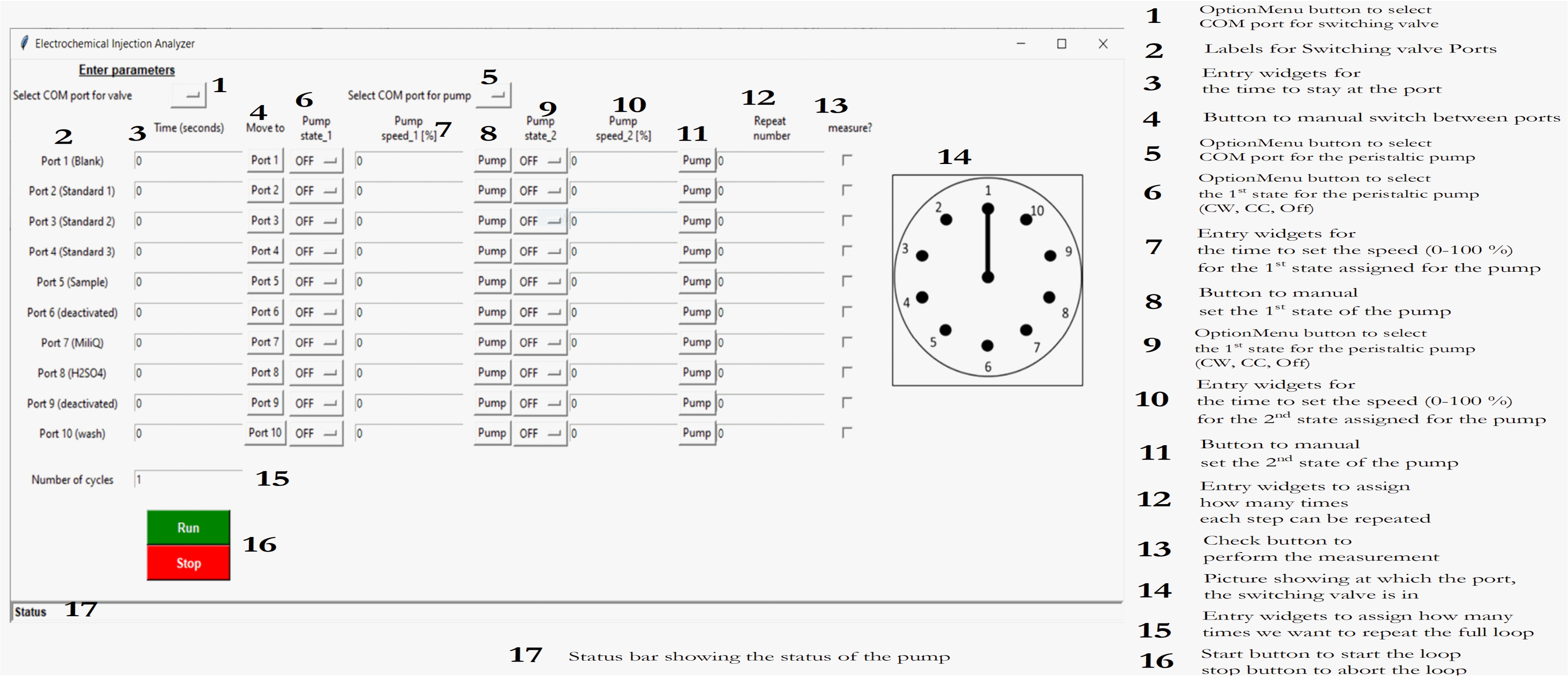

Figure S1 graphical user interface (GUI) of the 'Electrochemical FIA.exe' to program switching valve, peristaltic pump, and synchronization with the Metrohm µStat 400 Bi-potentiostat..

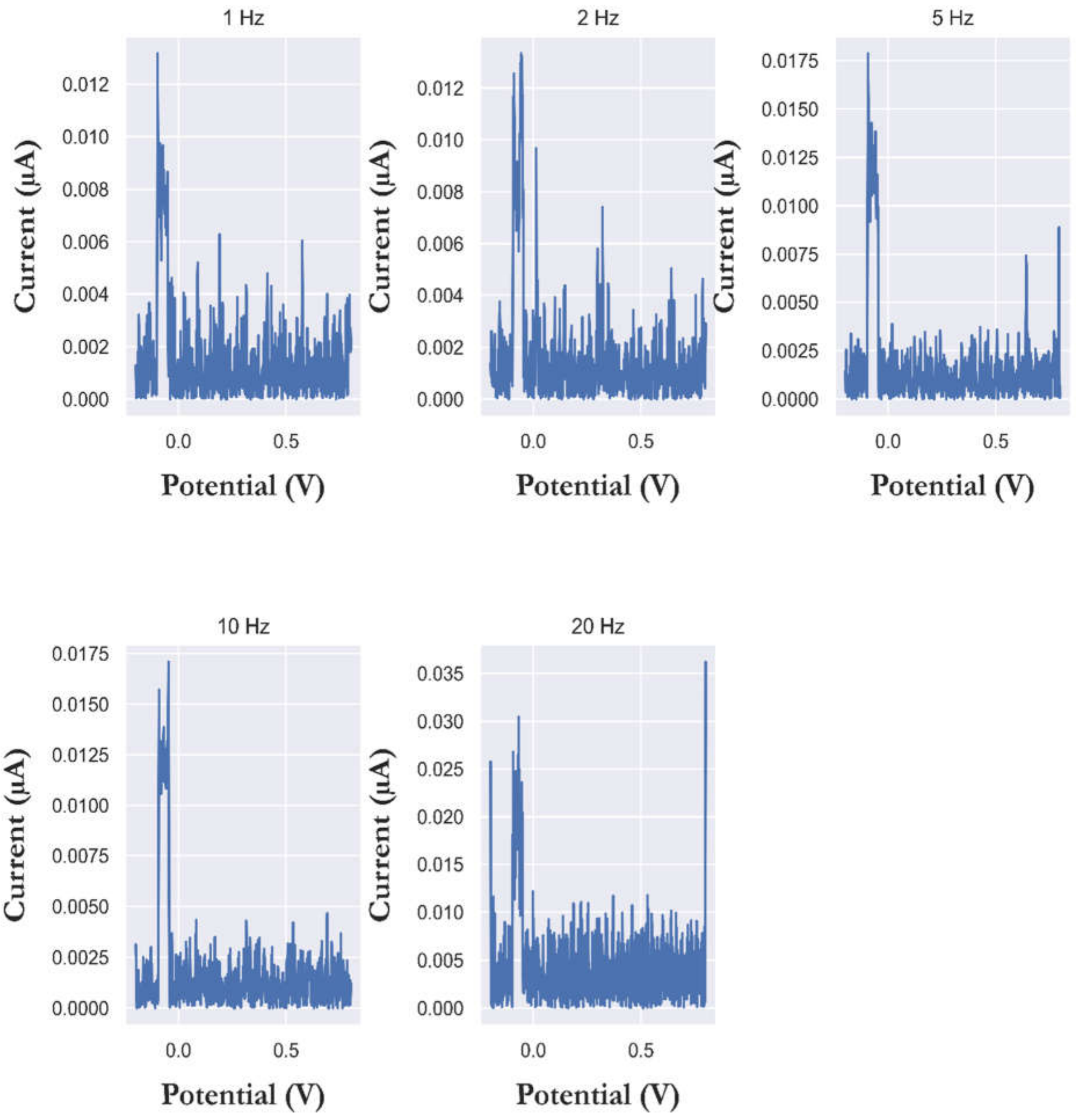

Figure S2 Square wave voltammograms of 0.5 μM PO_4^{3-} in 30 g/L NaCl (pH 0.8) (corrected voltammogram) at a step potential of 1 mV, square wave amplitude 25 mV at frequencies 1 Hz, 2 Hz, 5 Hz, 10 Hz and 20 Hz.

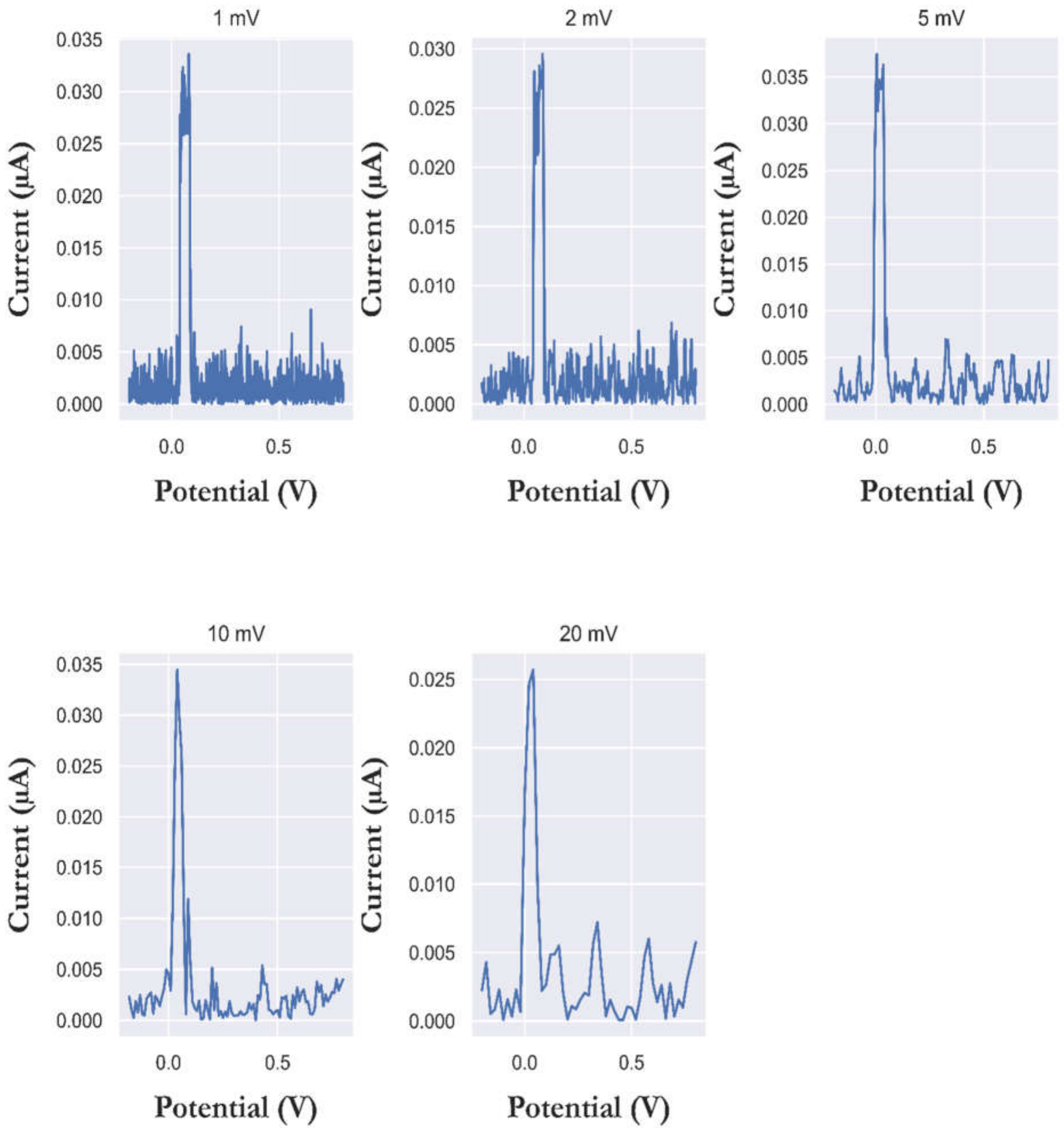

Figure S3 Square wave voltammograms of 0.5 µM PO_4^{3-} in 30 g/L NaCl (pH 0.8) (corrected voltammogram) at a frequency of 10 Hz, amplitude 25 mV at a step potential of 1 mV, 2 mV, 5 mV, 10 mV and 20 mV.

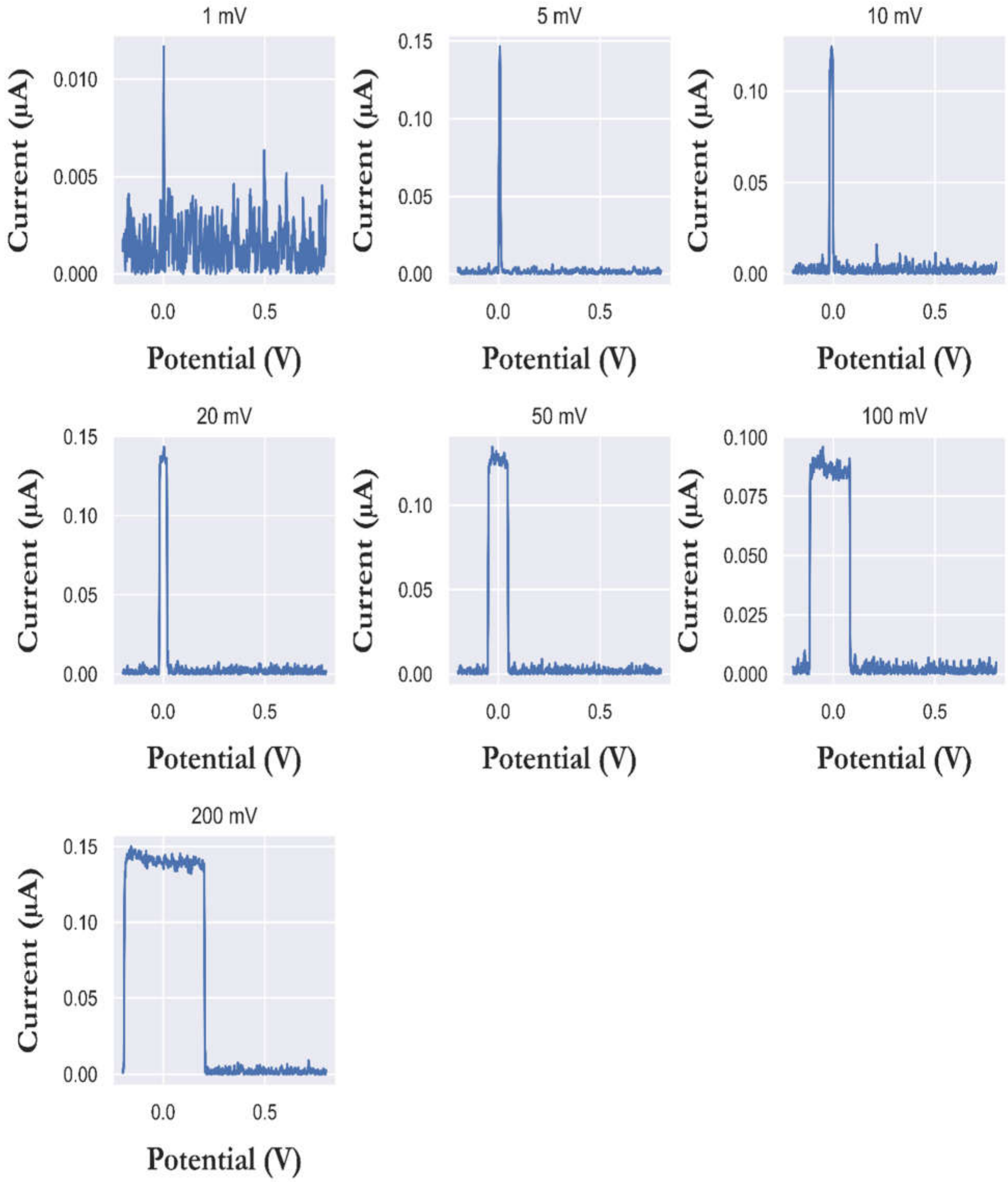

Figure S4 Square wave voltammograms of 0.5 µM PO_4^{3-} in 30 g/L NaCl (pH 0.8) (corrected voltammogram) at a step potential of 1 mV and a frequency of 10 Hz at square wave amplitudes of 1 mV, 5 mV, 10 mV, 20 mV, 50 mV, 100 mV and 200 mV.

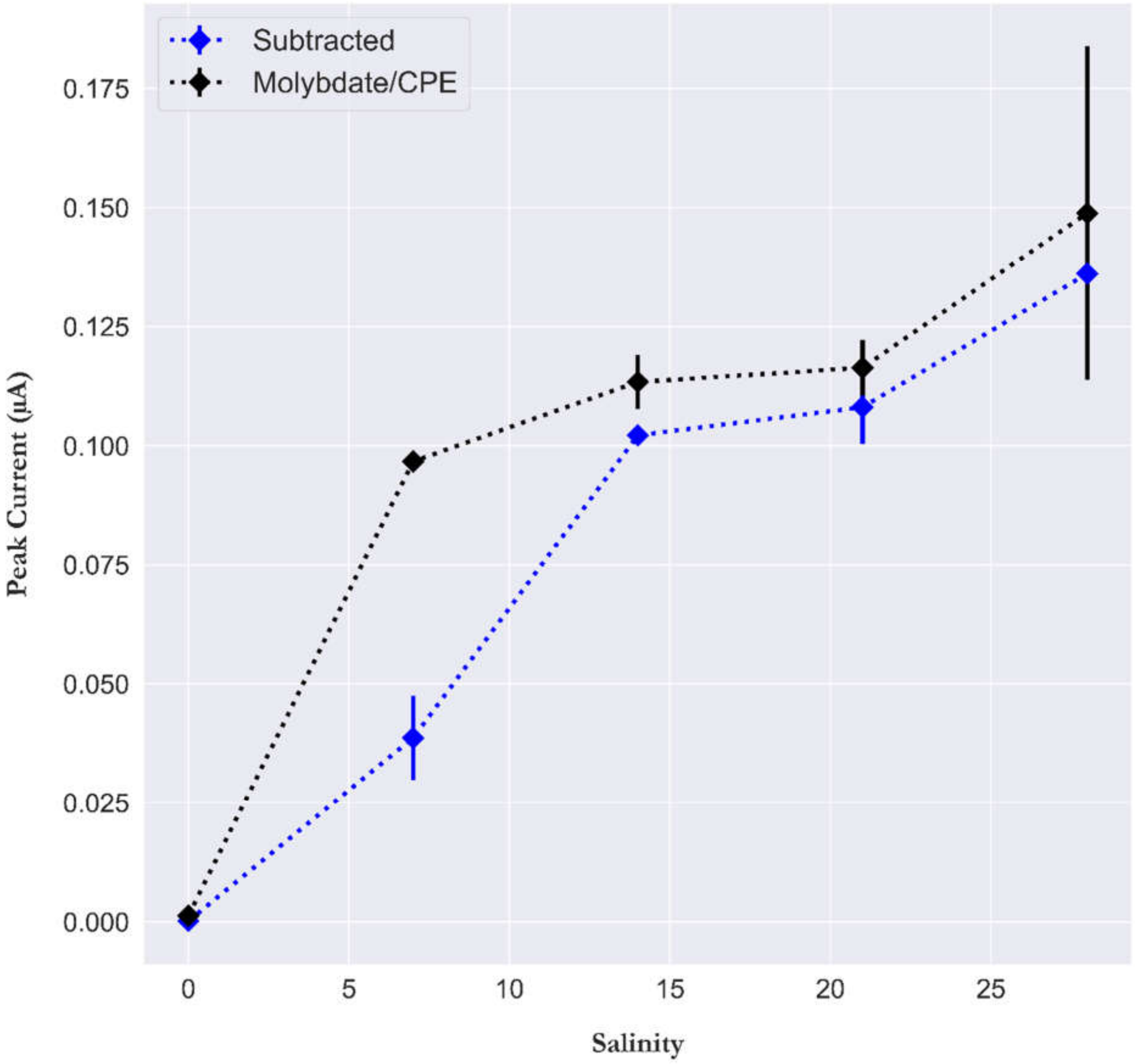

Figure S5 Effect of the variation of the salinity (0, 7, 14, 21 and 28) on the peak current of 1 μM PO_4^{3-} (pH 0.8) where the peak current of molybdate/CPE is shown as black circles and the peak current of corrected voltammogram is shown as blue circles. Error bar (n = 5).

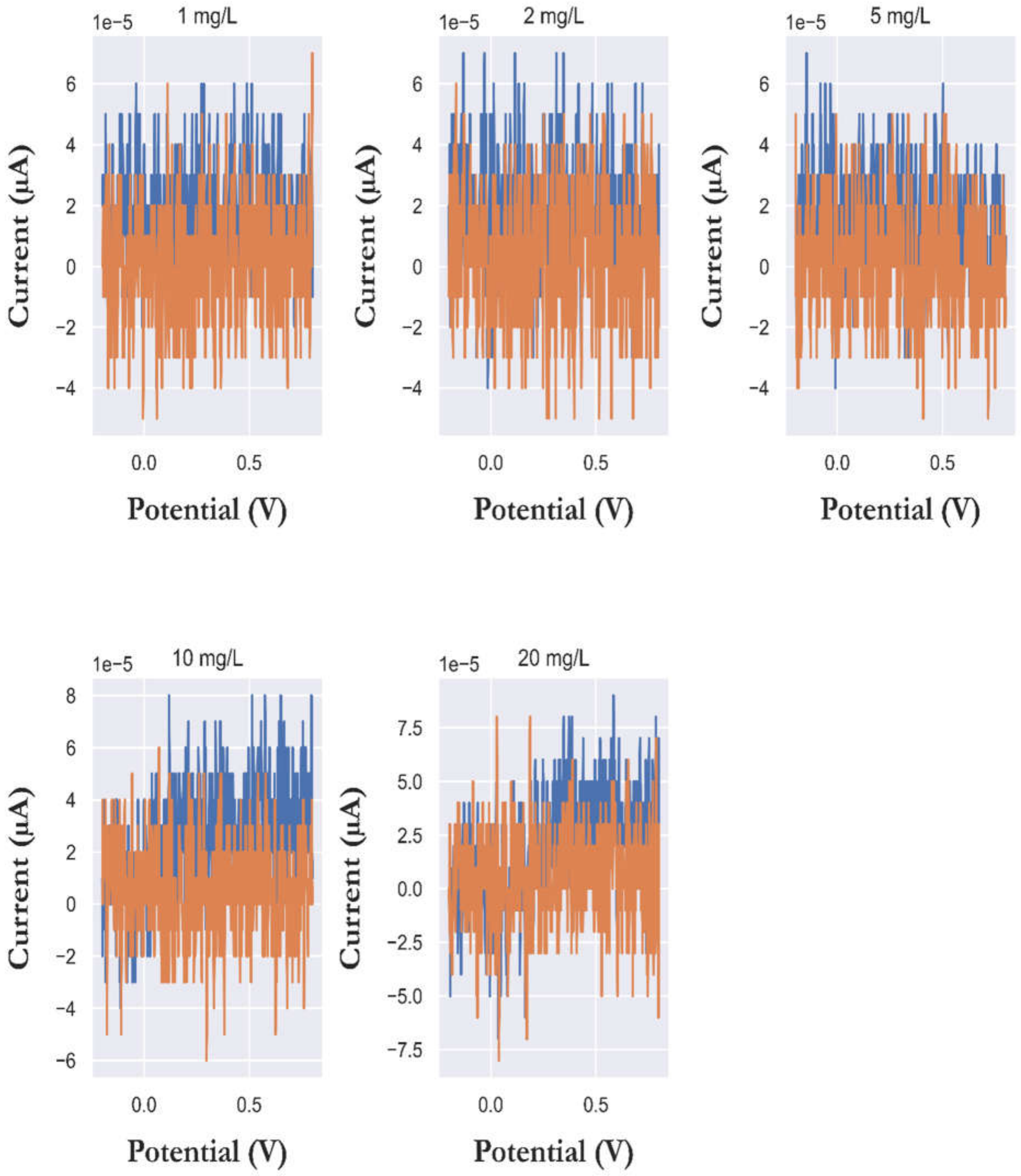

Figure S6 Square wave voltammograms of 1 μM PO_4^{3-} (30 g/L NaCl) pH 0.8 on molybdate/CPE (blue line) and CPE (orange line) in the presence of 1, 2, 5, 10, and 20 mg/L HA. Step potential 2 mV, frequency 10 Hz and amplitude of square wave 100 mV.

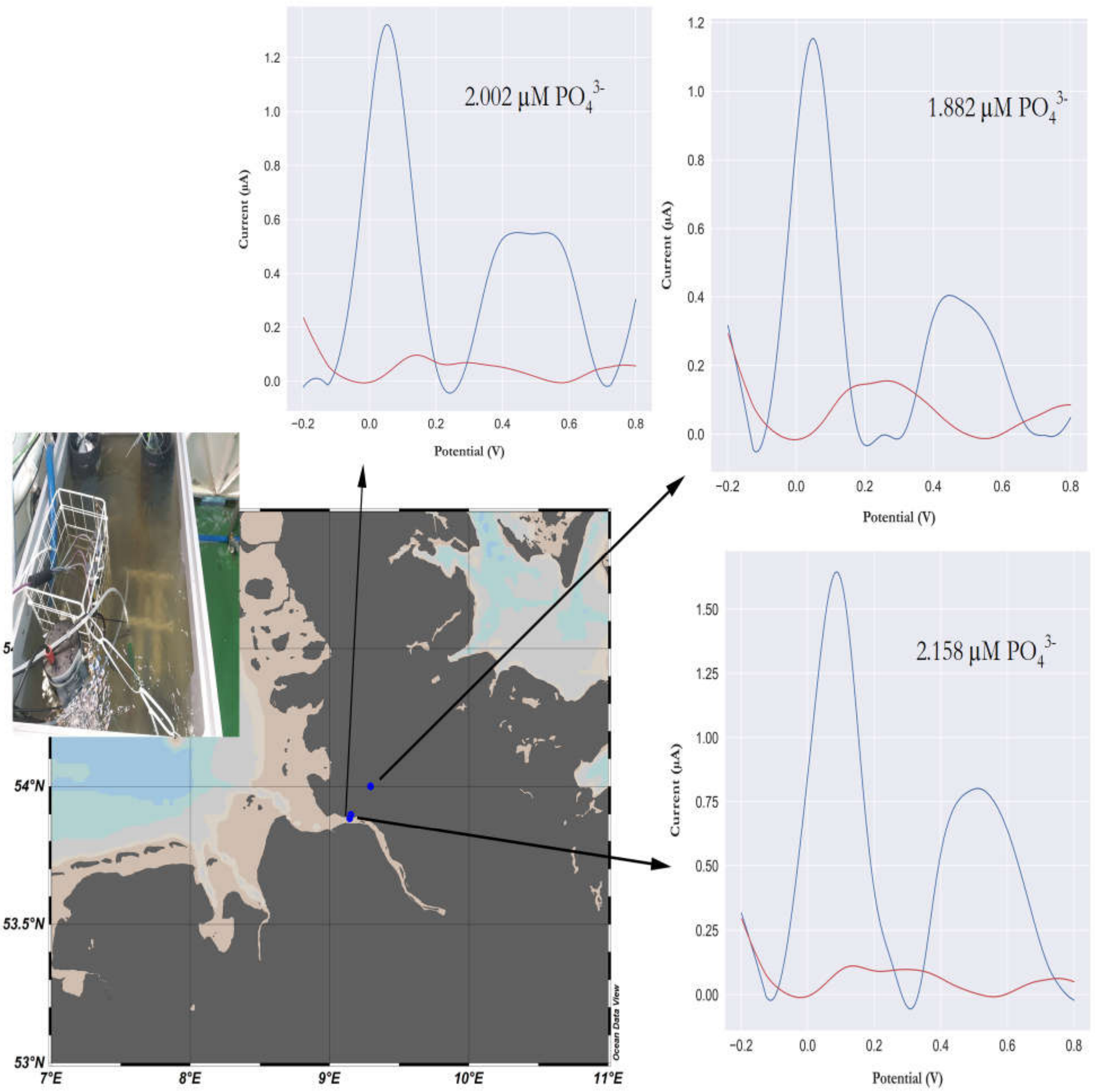

Figure S7 Location of three *on-site* data points taken at the Kiel Canal with square wave voltammograms at molybdate/CPE (blue line) and CPE (red line). The left inset shows the turbidity of the water entering the tank.

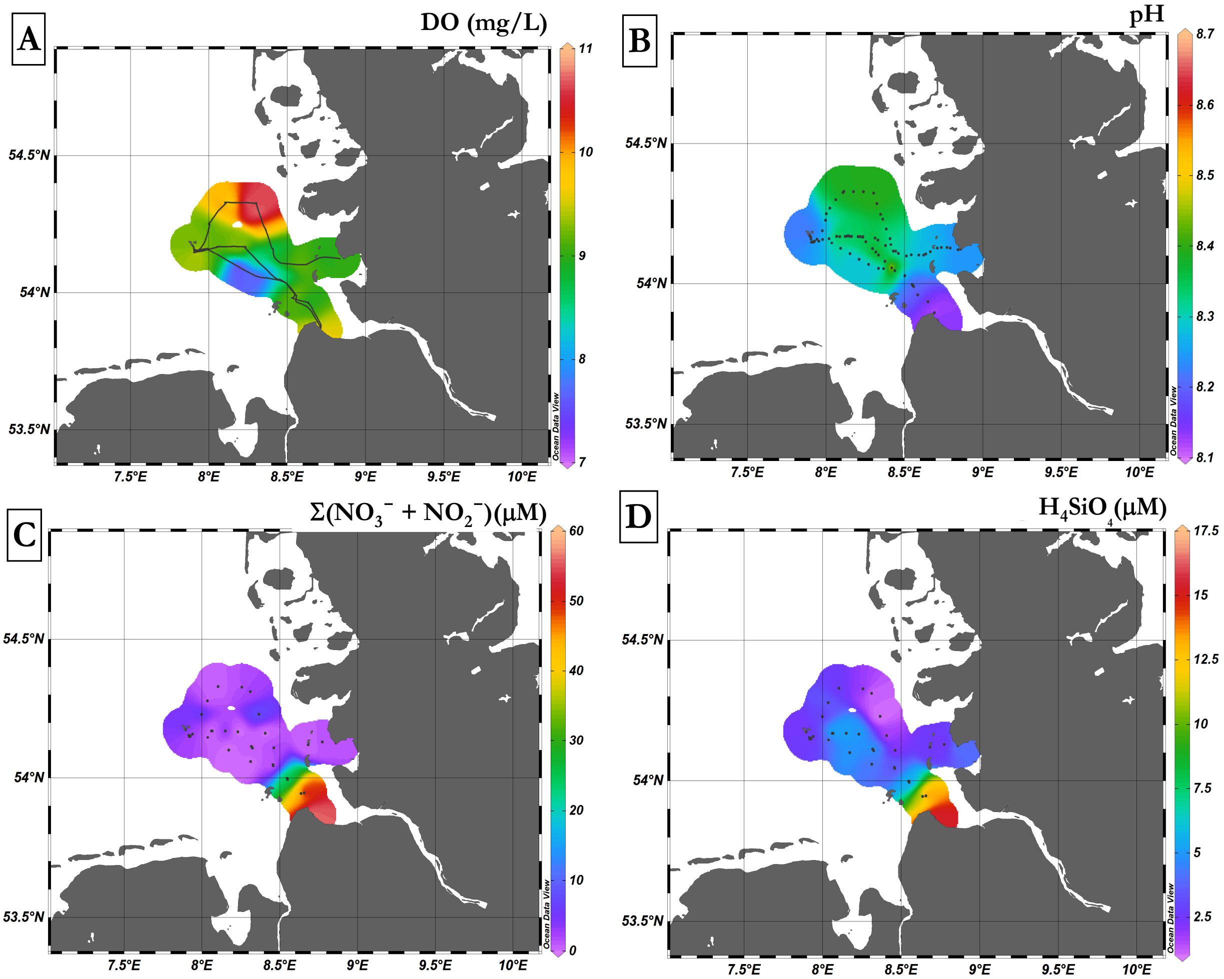

Figure S8 Overviews of (A) the distribution of surface dissolved oxygen (DO) in mg/L obtained from EXO sonde sensor, (B) distribution of pH obtained from sunburst SAMIpH and corrected for CTD salinity and temperature, (C) distribution of $\Sigma(NO_3^- + NO_2^-)$ in µM (bottom left panel) and (D) distribution of H_4SiO_4 in µM for the discrete samples collected from underway water supply and analyzed via QuAAtro air-segmented analyser.
